# Higher pulse frequency of near-infrared laser irradiation increases penetration depth for novel biomedical applications

**DOI:** 10.1371/journal.pone.0245350

**Published:** 2021-01-07

**Authors:** Ayan Barbora, Oryan Bohar, Ariel Alexander Sivan, Eyal Magory, Ariel Nause, Refael Minnes

**Affiliations:** Faculty of Natural Sciences, Department of Physics, Ariel University, Ariel, Israel; Massachusetts General Hospital, UNITED STATES

## Abstract

**Background:**

The clinical efficiency of laser treatments is limited by the low penetration of visible light used in certain procedures like photodynamic therapy (PDT). Second Harmonic Generation (SHG) PDT is an innovative technique to overcome this limitation that enables the use of Near Infrared (NIR) light instead of visible light. NIR frequency bands present an optical window for deeper penetration into biological tissue. In this research, we compare the penetration depths of 405 and 808 nm continuous wave (CW) lasers and 808 nm pulsed wave (PW) laser in two different modes (high and low frequency).

**Methods:**

Increasing thicknesses of beef and chicken tissue samples were irradiated under CW and PW lasers to determine penetration depths.

**Results:**

The 808 nm CW laser penetrates 2.3 and 2.4 times deeper than the 405 nm CW laser in beef and chicken samples, respectively. 808 nm PW (pulse frequency—500 Hz) penetrates deeper than CW laser at the same wavelength. Further, increasing the pulse frequency achieves higher penetration depths. High frequency 808 nm PW (pulse frequency—71.4 MHz) penetrates 7.4- and 6.0-times deeper than 405 nm CW laser in chicken and beef, respectively.

**Conclusions:**

The results demonstrate the higher penetration depths of high frequency PW laser compared to low frequency PW laser, CW laser of the same wavelength and CW laser with half the wavelength. The results indicate that integrating SHG in the PDT process along with pulsed NIR light may allow the treatment of 6–7 times bigger tumours than conventional PDT using blue light.

## Introduction

A significant contribution/application of laser medicine is Photodynamic therapy (PDT) which uses light and a chemical substance called photosensitizer (PS) to generate molecular oxygen and elicit cell death. This phenomenon of phototoxicity has been a promising development with various applications in microbial detection and treatment of infections [1,2] involving even antibiotic resistant bacteria [3], enhanced wound healing [4] and cancer therapy [5]. As a clinically approved minimally invasive therapeutic procedure PDT exerts a selective cytotoxic activity toward malignant cells with demonstrated effectivity in cancers [6,7]—of the skin, head and neck, digestive system [8], urinary system [9], lungs, etc. PDT results in significantly reduced tumour weights compared to untreated samples [5], reducing the number of clonogenic tumour cells. However, its clinical effectivity remains limited to treatment of small tumours [6]. Various technical aspects of the PDT treatment are accountable for the limited effect of PDT in treating large tumours [10,11] with penetration depth of radiation used being a key contributory factor.

Conventional PDT employs the visible electromagnetic (EM) spectrum within the range of 400–750 nm [12,13] to excite the corresponding photosensitisers. However, penetration of visible light through biological tissue is very limited. While red light can penetrate about 6 mm beneath the surface of the skin, blue light penetrates barely 1 mm into tissue [14]. It was shown that the transmittance of visible light through biological tissue increases with increasing wavelength [15,16]. Measurements of light intensity transmittance have demonstrated that the optical penetration depth in biological tissue does not depend on the optical power [17]. Light has its maximum depth of penetration in tissue within the range of 750–950 nm termed as the Near-infrared (NIR) window [18], which has been effectively used for optical imaging in live animals [19]. Thus, a system utilising this band is expected to be more effective in PDT treatment [20,21].

Power transmission in Low Level Laser Therapy (LLLT) decreases with increasing tissue thickness of skin, fat, and muscles [22]. Tissue transmission associated attenuation coefficient was the highest for 660 nm, minimal for 830 nm and the least for 904 nm [22]. Optical transmission of controllable infrared source parameters demonstrates maximum efficiencies of 86% [23] with higher duty cycles transmitting greater power over shorter exposure times while lower duty cycles transmit higher power at lower temperatures. Variations in efficiency of transmission indicate that the power transmission percentage can be appropriately tuned.

Second Harmonic Generation (SHG) is an optoelectronic process mediated by nonlinear material which produces photon up conversion by frequency doubling (or wavelength halving). SHG can be used to allow NIR light to generate visible light of appropriate wavelength to excite PSs and produce the photodynamic effect [24]. This technique overcomes the low penetration depth of visible light while allowing the same photosensitisers to be used effectively in conjunction with NIR light.

NIR light in the range 770–850 nm, projected transcutaneous in Sprague–Dawley rats demonstrated the highest transmission (penetration) through all overlying tissue layers of the spinal cord and blood. 810 nm laser with 150 mW incident power translated into 9 mW reaching the spinal cord through overlying thickness of 18 mm (6% power transmission) [25]. Light at 808 nm penetrates as much as 54% deeper than 980 nm light [26]. In bovine tissue, light at 808 nm with an Initial Power Density (IPD) of 1 W/cm^2^ attenuates over a depth of 3.4 cm delivering 1 mW/cm^2^ in situ [26]. Hence, NIR and especially 808 nm is expected to increase the efficacy of PDT.

Pulsed wave (PW) laser irradiation provides added advantages over continuous wave (CW) lasers. The high instantaneous power fluxes in PW provide efficient energy transfer [27] and the interval between pulses allows for heat dissipation to avoid thermal ablation [28].

In this study we investigated the penetration depths of 808 nm PW in two different modes; high frequency (HF) and low frequency (LF), 808 nm CW laser and 405 nm (the corresponding wavelength for direct excitation of the PS) CW laser using different meat samples representing different kinds of tissue.

## Materials and methods

### Sample preparation

**Fig 1:**
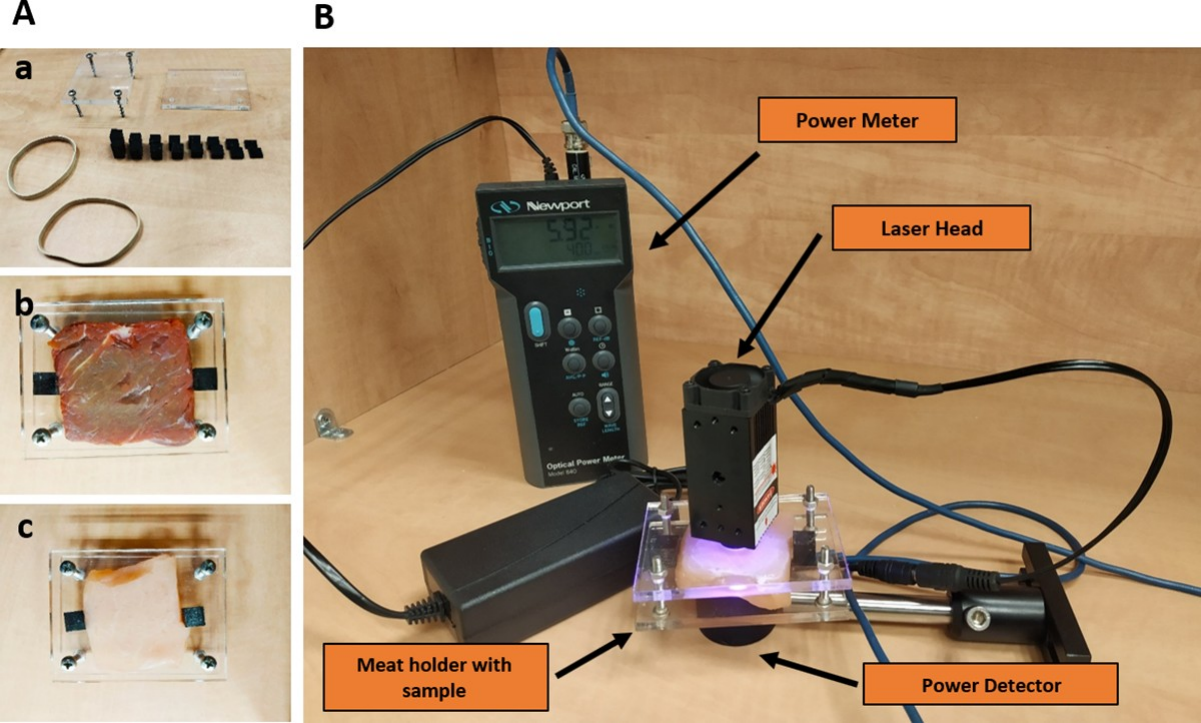
Experiment setup. **A** (a) Acrylic glass meat holder and spacers with (b) beef or (c) chicken samples. **B** Irradiation setup to measure penetration depth.

The Beef (rib) and Chicken (breast) samples were purchase from "Campus Market" (Building 102, 27 Ramat Hagolan Street, Ariel). The samples were kept frozen and the amount needed was thawed to room temperature before each experiment. Beef meat is darker than chicken meat (The appearance of the samples can be seen in Fig 1) due to higher protein content [29]. This contrast was used to represent different types of human internal tissues like muscles and non-muscles respectively for the purpose of measuring penetration depths. Sliced sections of either Beef or Chicken (measuring 2 mm, 4 mm, 6 mm, 8 mm, 10 mm, 12 mm, 14 mm and 18 mm. Thickness error: ±0.2 mm) were placed within a screw-in sample holder made of acrylic glass (providing optical transparency in the bandwidth of the spectrum investigated) with appropriate spacers (Fig 1).

### Sample irradiation

Samples placed within the acrylic glass meat holder were irradiated with an 808 nm and 405 nm continuous diode lasers and 808 nm PW (Astrella by Coherent) which is normally used to drive a photo-cathode RF e-gun [30]. We used the PW in two different modes: LF (repetition rate—500 Hz, pulse duration—50 fs, spot diameter—11 mm) and HF (repetition rate—71.4 MHz, pulse duration—5 fs, spot diameter—3 mm). The average power, peak power, energy per pulse, and power density of the LF and HF pulsed laser were 2.55 W, 102 GW, 5.1 mJ, 2.68 W/cm^2^ and 216 mW, 605 W, 3.0 nJ, 3.06 W/cm^2^ respectively. In the case of the 404 nm and 808 nm continuous lasers average powers were 135 mW and 165 mW respectively. The parameters of the lasers used for the irradiation of the samples are summarized in Table 1. Transmitted powers were measured before (transmittance through blank acrylic glass holder—*I*_*0*_) and after (transmittance through acrylic glass holder with meat/chicken slices—*I*) sample insertion and normalised to those of blank acrylic glass meat holder for stringency of analysis. Power measurements were done using a Newport optical power meter (Model 840) and Newport 883SL photodiode sensor (OD 3) for the CW lasers and Coherent power detector/sensor (OD-COHERENT PowerMax—USB PM10) for the PW.

**Table 1:** Lasers' parameters.

	Pulse frequency	Power density	Peak power	Energy per pulse	Average power
**CW 1**	N/A	N/A	N/A	N/A	135 mW
**CW 2**	N/A	N/A	N/A	N/A	165 mW
**PW LF**	500 Hz	2.68 W/cm^2^	102 GW	5.1 mJ	2.55 W
**PW HF**	71.4 MHz	3.06 mW/cm^2^	605 W	3.0 nJ	216 mW

Parameters of the lasers used for irradiation of the samples. **CW 1**–405 nm; **CW 2**–808 nm; **PW LF**—808 nm, 50 fs; **PW HF**—808 nm, 5 fs.

### Penetration depth analysis

Beer-Lambert law explaining the exponential attenuation of electromagnetic spectra transmitted through tissue was used to compute the optical penetration depth. Using this law, transmittance (*T*) as a ratio of the transmitted light (*I*) to the incident light (*I*_*0*_) is derived by the equation:

$$T=\frac{I}{I_{0}}=e^{-\mu d}$$

where *μ* is the attenuation coefficient and *d* is the thickness of the tissue sample. Penetration depth (*δ*) is defined as the depth at which the transmittance inside the tissue equals “1/*e*”. Therefore, transmittance can be expressed in terms of the penetration depth as:

T=e−dδ

For each laser, transmittances have been plotted as a function of tissue thickness. Each point in the graph is an average of 2–3 measurements. Each graph has been fitted to the function of e−xδ to get the penetration depth (*δ*) and error (Δ*δ*). The least squares analysis was done using MATLAB R2019a software.

## Results

### 808 nm laser penetrates deeper than 405 nm under continuous wave irradiation

**Fig 2:**
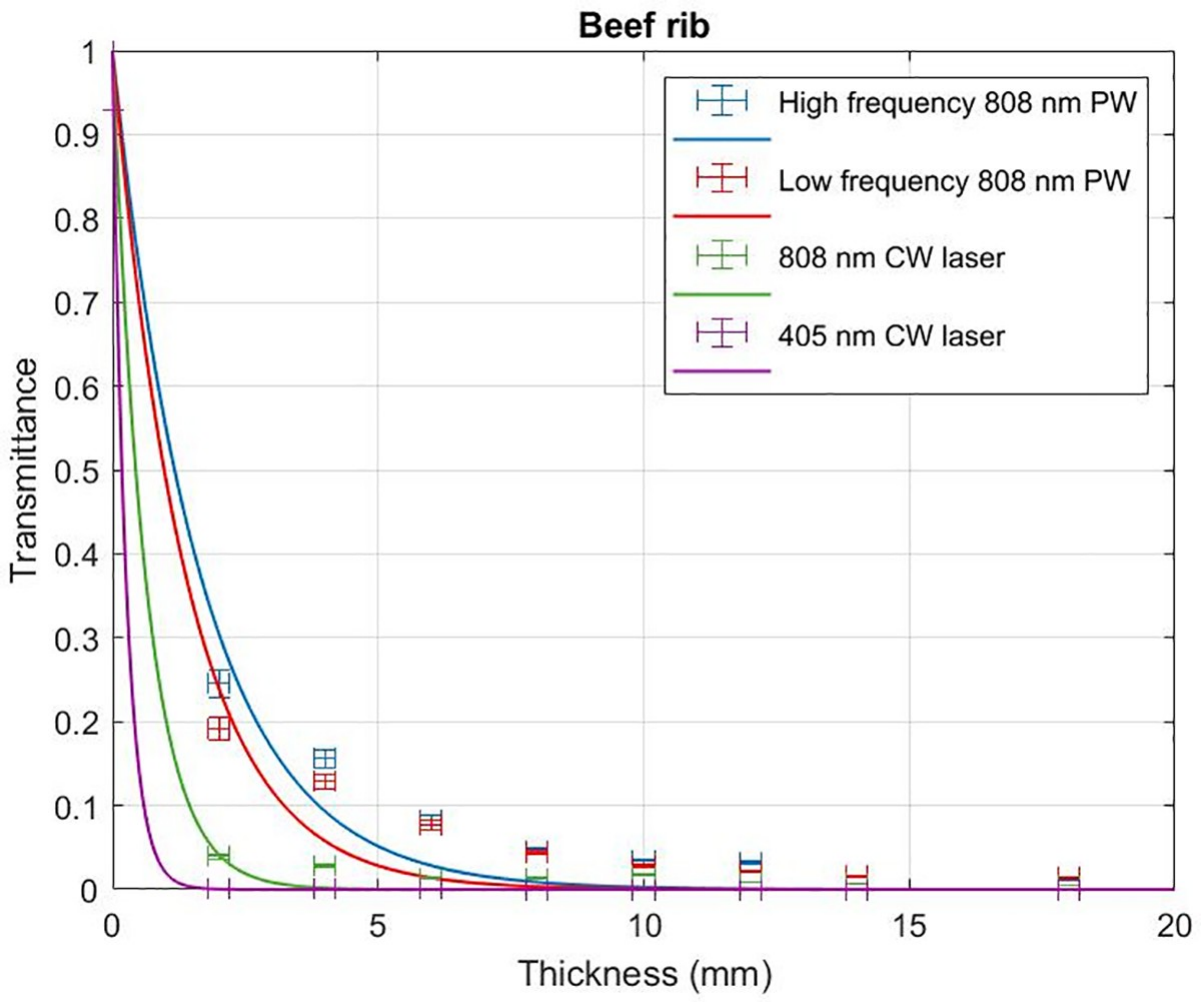
Transmittance through beef rib samples. Transmittance of 405 nm CW laser, 808 nm CW laser irradiation, low frequency 808 nm PW and high frequency 808 nm PW with varying thickness of beef samples.

**Fig 3:**
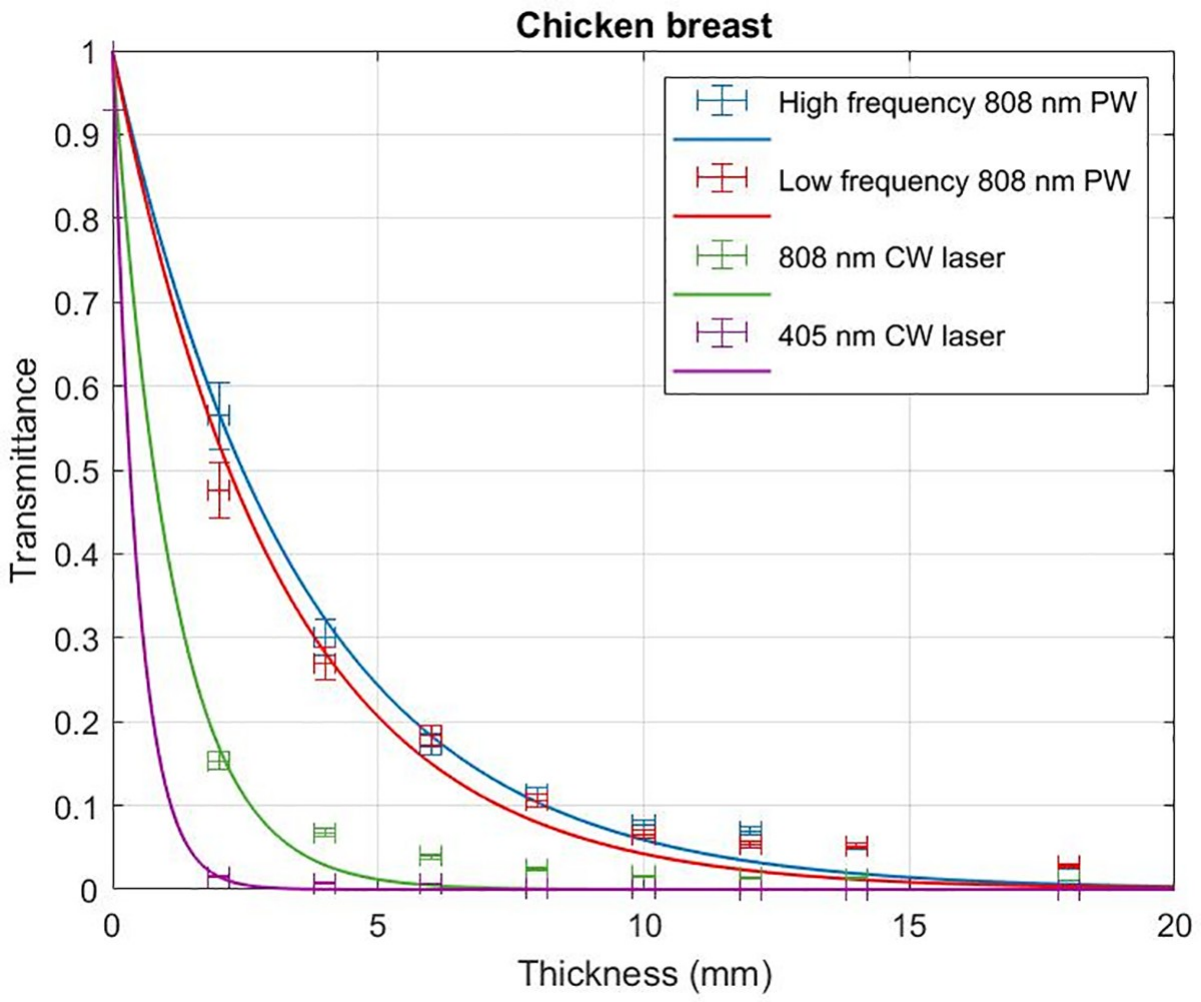
Transmittance through chicken breast samples. Transmittance of 405 nm CW laser, 808 nm CW laser irradiation, low frequency 808 nm PW and high frequency 808 nm PW with varying thickness of chicken samples.

Beef and chicken samples were irradiated with 405 nm and 808 nm CW lasers at 135 mW and 165 mW incident powers respectively and the associated transmittance with varying levels of tissue thickness analysed. 405 nm CW laser reaches a penetration depth of 0.28 ± 0.03 mm in beef samples (Fig 2) while 808 nm CW laser reaches a penetration depth of 0.63 ± 0.16 mm. In chicken samples 405 nm CW laser reaches a penetration depth of 0.48 ± 0.07 mm (Fig 3) while 808 nm CW laser reaches a penetration depth of 1.13 ± 0.21 mm.

### 808 nm pulsed laser irradiation penetrates deeper than continuous wave irradiation

Beef and chicken samples were irradiated with pulsed 808 nm laser (500 Hz repetition rate, 50 fs pulse width) and the associated transmittance with varying levels of tissue thickness were compared with that of the CW irradiation. Pulsed 808 nm reached a penetration depth of 1.41 ± 0.41 mm in beef samples (Fig 2) while CW 808 nm reached a lower penetration depth of 0.63 ± 0.16 mm. In chicken samples pulsed 808 nm reached a penetration depth of 3.24 ± 0.48 mm (Fig 3) while CW 808 nm reached a lower penetration depth of 1.13 ± 0.21 mm.

### High frequency PW irradiation penetrates deeper than low frequency pulsed laser irradiation

Beef and chicken samples were irradiated with HF 808 nm PW (71.4 MHz, 5 fs) and the associated transmittance with varying levels of tissue thickness were compared with that of the LF 808 nm PW (500 Hz, 50 fs) irradiation. HF pulsed 808 nm reached a penetration depth of 1.70 ± 0.43 mm in beef samples (Fig 2) while LF pulsed 808 nm reached a lower penetration depth of 1.41 ± 0.41 mm. In chicken samples HF pulsed 808 nm reached a penetration depth of 3.56 ± 0.34 mm (Fig 3) while LF pulsed 808 nm reached a lower penetration depth of 3.24 ± 0.48 mm. Table 2 summarizes all the measured penetration depths.

**Table 2:** Penetration depths.

	Chicken breast	Beef rib
**CW 1**	0.48 ± 0.07 mm	0.28 ± 0.03 mm
**CW 2**	1.13 ± 0.21 mm	0.63 ± 0.16 mm
**PW LF**	3.24 ± 0.48 mm	1.41 ± 0.41 mm
**PW HF**	3.56 ± 0.34 mm	1.70 ± 0.43 mm

Penetration depth values based on fitted parameters from the figures. **CW 1**–405 nm, 135 mW CW laser; **CW 2**–808 nm, 165 mW CW laser; **PW LF**—808 nm, 50 fs, 500 Hz, 2.55W PW laser; **PW HF**—808 nm, 5 fs, 71.4 MHz, 216 mW PW laser.

## Discussion

As discussed in the introduction, the power of the laser does not affect the penetration depth [14]. Therefore, differences in penetration depth must be related to the wavelength, laser type (CW or PW) and the frequency of the PW. The experiments demonstrate that transmittance of the CW laser passing through chicken samples is 2-times greater on average than through beef samples. Chicken samples are of a lighter color compared to beef accountable to a higher myoglobin content in the latter [29]. Further, transmittance of both high and LF type pulsed lasers passing through chicken samples are also about 2-times greater than through beef samples. This observation corroborates with the trend of increased transmission as one moves from mainly muscular tissue to mainly fatty tissue [31].

The results from CW laser irradiation consistently show that the penetration of NIR 808 nm wavelength (0.63 ± 0.16 mm in beef and 1.13 ± 0.21 mm in chicken) is more than twice higher than the penetration of visible range 405 nm wavelength (0.28 ± 0.03 mm in beef and 0.48 ± 0.07 mm in chicken). Transmittance of light through any specific thickness of tissue improves when a PW is used in contrast to a CW laser, operating at the same wavelength of 808nm. For beef rib samples, 808nm PW–HF and LF irradiation resulted in 2.7- and 2.2-times improved transmittance than that of the CW respectively. For chicken breast, 808nm PW–HF and LF irradiation resulted in 3.2- and 2.9-times improved transmittance than that of the CW respectively.

HF 808 nm PW irradiation resulted in a 20% increase in transmittance through beef rib samples with respect to LF 808 nm PW exposure. In chicken samples, transmittance increased by 10% when HF pulsed irradiation replaced the LF pulsed exposure at 808 nm. HF 808 nm PW penetrates 7.4- and 6.0-times deeper than 405 nm CW laser in chicken and beef, respectively. Increasing diameter of laser irradiation beams are known to account for greater penetration. However, in this case our experiments involved a 3 mm spot diameter for the HF laser and a 11 mm spot diameter for the LF laser (ref. Methods).

A possible explanation for the results is that the high photon density of the PW laser (compare to the CW laser) excites a large percentage of electrons in the sample (close to saturation), rendering the irradiated zone transiently transparent for the rest of the incoming photons. This phenomenon is also indicated by the observed increase in transmission associated with increasing the pulse frequency in the PW regimes. For both HF and LF the pulse duration (5 fs and 50 fs, respectively) is shorter than the lifetime of the excited state, estimated as 10^−7^ to 10^−5^ s. However, the difference in the repetition rates between the two modes might affect the percentage of the excited electrons in the irradiation area. The low repetition rate (time between pulses = 2 ms) of the LF laser guarantees that the next pulse will reach the sample only after all the excited electrons returned to the ground state. However, due to the high repetition rate (time between pulses = 14 ns) of the HF laser the next pulse will reach the sample while the electrons, excited by the previous pulse, are still in the excited state. This will accumulate electrons in the excited state leading to higher transient transparency as compared to the LF irradiation.

The 4 main NIR optical windows useful for biomedical imaging and therapeutics are classified into bands from—750 to 950 nm, 1100 to 1350 nm, 1600 to 1870 nm and 2100 to 2200 nm [32]. The first window presents the lowest levels of absorption by tissue water as compared to the remaining three bands. Despite this property, often image quality is reduced due to stronger absorption peaks from lipids, hemoglobin and deoxyhemoglobin resulting in blurring due to the molecular process of Rayleigh/Mie scattering [33]. This has necessitated development of special longer NIR wavelength CCD detectors and equipment to overcome the described problems [32] in order to utilize the second, third and fourth optical windows. In contrast, our results demonstrate that increased tissue transparency can be achieved by simply using higher pulse frequencies within the first NIR optical window itself allowing deeper penetration and ease of application.

Scattering constitutes a dominant factor determining tissue penetration within the NIR optical windows [34] with absorbed photons getting trapped in the tissue while other scattered photons either get absorbed or scattered and are detectable at appropriate distance(s) from the incident light source. However, scattering exhibiting a weak dependence on wavelength; the effective use of the NIR optical windows are primarily limited by absorption of blood at shorter wavelengths and water at longer wavelengths [33]. The common minimum absorption trough of the major tissue components (water, deoxygenated and oxygenated blood, melanin, etc.) lies within the 650–900 nm bandwidth [34] which influence the in-vivo optical properties of tissues pertaining to PDT treatment(s). However, due to the large variations of the absorption and scattering parameters biological tissue are not uniformly absorbing and scattering objects [35]. As described in the discussion the higher repetition rate (time between pulses) of the HF laser allows each subsequent pulse to reach the sample while the electrons excited by the previous pulse are still in the higher energy state, enabling an accumulation of excited state electrons leading to transient transparency as compared to the LF irradiation. This phenomenon along with the higher number of photons being delivered by the HF laser overcomes the limits of scattering dependent interactions [34] providing the improved penetration depths as demonstrated by our experiments.

Our experiments demonstrate that 808 nm lasers penetrate deeper than 405 nm CW laser for any given tissue. Irradiation of biological tissue(s) by lasers result in four main types of interactions–reflection, absorption, scattering and transmission [36]. Pogue et al reported some greater effective penetration for pulsed light, but they attributed this to transient alteration of absorption by the PSs [37]. In contrast, our experiments involved no PSs. The results demonstrate a novel discovery that NIR PW irradiation improves the associated penetration depth significantly in contrast to CW exposures. In addition, higher pulse frequency (repetition rate) further enhances the penetration depth compare to lower frequency of the same wavelength. This phenomenon encourages further investigations for novel processes of laser tissue interactions and related applications [36].

## Conclusions

Laser power transmission associated attenuation for cancerous tissues being lower than that of normal tissues over all the 4 NIR optical windows [32], our results suggest that adoption of higher pulse frequency irradiation regimes can effectively overcome the penetration depth limitation of using PDT for treating large tumors [10,11].

In the emerging field of nanomedical imaging and laser mediated therapies PEGylated nanoparticles [38] and organic/inorganic nanohybrids [39] have improved drug delivery and circulation to targeted sites [40]. This has allowed for improvements in laser-based PDT. However, PDT is still limited to small tumors due to the penetration depth of the excited light. Besides overcoming the limited penetration depths of visible light, PDT mediated by NIR light also enables development of optoelectronic applications to improve clinical efficiency. SHG allows for conventional photosensitizers (usually operating under visible light) to be used with greater clinical efficiency [24,41] enabling the use of PDT for bigger tumors. Our results indicate that integrating SHG in the PDT process [24] will allow the excitation of PSs with NIR light pulsed appropriately as demonstrated by our experiments reaching 6–7 times higher penetration depths than conventional blue light. In addition, recent developments of using NIR absorbing chromophores [18] like BODIPY, cyanine, porphyrin, phthalocyanine, nano assemblies and etc., applied in conjunction to experimental indications of this study will enhance clinical performance of the photodynamic mechanism for selective ablation of tumors and enabling targeting of sub-cellular organelles. Clinical adaptations of these results which achieve greater penetration than conventional standards are expected to also improve applications of PDT for reducing microbial loads in dermatology [42] and in treatment of emerging diseases like COVID-19 [43].
